# The Role of Flexibility and Conformational Selection in the Binding Promiscuity of PDZ Domains

**DOI:** 10.1371/journal.pcbi.1002749

**Published:** 2012-11-01

**Authors:** Márton Münz, Jotun Hein, Philip C. Biggin

**Affiliations:** 1 Structural Bioinformatics and Computational Biochemistry, University of Oxford, Oxford, United Kingdom; 2 Department of Statistics, University of Oxford, Oxford, United Kingdom; 3 Oxford Centre for Integrative Systems Biology, Department of Biochemistry, Oxford, United Kingdom; Florida State University, United States of America

## Abstract

In molecular recognition, it is often the case that ligand binding is coupled to conformational change in one or both of the binding partners. Two hypotheses describe the limiting cases involved; the first is the induced fit and the second is the conformational selection model. The conformational selection model requires that the protein adopts conformations that are similar to the ligand-bound conformation in the absence of ligand, whilst the induced-fit model predicts that the ligand-bound conformation of the protein is only accessible when the ligand is actually bound. The flexibility of the apo protein clearly plays a major role in these interpretations. For many proteins involved in signaling pathways there is the added complication that they are often promiscuous in that they are capable of binding to different ligand partners. The relationship between protein flexibility and promiscuity is an area of active research and is perhaps best exemplified by the PDZ domain family of proteins. In this study we use molecular dynamics simulations to examine the relationship between flexibility and promiscuity in five PDZ domains: the human Dvl2 (Dishevelled-2) PDZ domain, the human Erbin PDZ domain, the PDZ1 domain of InaD (inactivation no after-potential D protein) from fruit fly, the PDZ7 domain of GRIP1 (glutamate receptor interacting protein 1) from rat and the PDZ2 domain of PTP-BL (protein tyrosine phosphatase) from mouse. We show that despite their high structural similarity, the PDZ binding sites have significantly different dynamics. Importantly, the degree of binding pocket flexibility was found to be closely related to the various characteristics of peptide binding specificity and promiscuity of the five PDZ domains. Our findings suggest that the intrinsic motions of the apo structures play a key role in distinguishing functional properties of different PDZ domains and allow us to make predictions that can be experimentally tested.

## Author Summary

Proteins that are capable of binding to many different ligands are said to have broad specificity. This is sometimes also referred to as promiscuity. Whether a protein is promiscuous or not can sometimes be readily explained by the structure of the protein and the ligand in terms of electrostatic and steric effects. Sometimes however, this simple interpretation can struggle to explain the experimentally observed data. A prominent case in point is the PDZ domains. These small protein domains bind to unstructured regions of other proteins and are involved in many signaling pathways. Some PDZ domains appear to be more promiscuous than others, but this has been difficult to explain purely on the basis of the composition of residues in the binding groove. In this work we examine the dynamics and conformational flexibility of five key PDZ domains and demonstrate that despite similar folds, these proteins can exhibit quite different dynamics. Furthermore the difference in the dynamic behavior appears to correlate with the observed promiscuity. Our findings suggest that knowledge of the dynamic behavior of the PDZs can be used to rationalize the extent of expected promiscuity. Such knowledge will be critical for drug design against PDZ domains.

## Introduction

A number of structural studies comparing holo and apo forms of proteins have demonstrated that ligand binding is often coupled to conformational changes of the interacting partners [1]–[3]. The real challenge is, however, to uncover the exact sequence of events resulting in the observed structural changes. Two main models, the induced fit (Koshland) and the conformational selection (or population shift) hypothesis (see [4] for a review), have been introduced to describe the limiting cases of the complex process of molecular recognition [5]–[8].

According to the induced fit model, ligand binding happens first and the formation of a ‘weak complex’ is followed by the conformational rearrangement of the protein that results in stronger binding [9]. By contrast, in the conformational selection model, the intrinsic dynamics of the protein lead it to spontaneously transition between a stable unbound and a less stable ‘bound conformation’. As the apo protein actually visits the bound state with significant probability, the ligand can bind directly to this conformation shifting the distribution of conformers towards the bound population. As recently reviewed [4], it seems likely that the induced fit and conformational selection mechanisms often act together in the ligand recognition process.

Furthermore, in terms of protein-protein interactions, it is increasingly clear that many proteins display functional promiscuity which requires them to be able to interact with multiple partners [10]. If the conformational selection mechanism is involved in promiscuous ligand binding, this assumes that the protein needs to visit multiple (often dissimilar) binding conformers capable of binding the different ligands. An example of structural evidence of such multi-specificity can be found in the X-ray crystallography study of the SPE7 antibody (a monoclonal immunoglobulin E raised against a 2,4-dinitrophenyl hapten) that has been shown to adopt different binding conformers and is consequently able to bind to multiple unrelated antigens [11]. Another example is the NMR study of apo ubiquitin which has found that this protein exists in an ensemble of conformers that are almost identical to the conformations of ubiquitin in complex with 46 different binding partner proteins [12]. One of the best known examples of a promiscuous enzyme is cytochrome P450 which has been shown to adopt a great variety of active site conformations and is able to bind and transform diverse substrates [13].

As shown by these examples, the intrinsic dynamics of promiscuous proteins let them visit multiple unrelated binding conformers and the property of multispecificity seems to be related to conformational flexibility. Promiscuous proteins that are able to bind to multiple partners through conformational selection need to explore a larger conformational space than those that bind to only a single partner. More rigid binding sites therefore may have restricted specificity with the benefit of higher binding affinity.

Indeed, a study of human cytochrome P450 enzymes has found that while a relatively rigid member of the family (CYP2A6) has narrow substrate specificity, the most flexible member (CYP3A4) is also the most promiscuous one [14]. Functionally promiscuous proteins could be of key importance for the emergence of new functions in protein evolution. Recent research about the relationship between binding promiscuity, conformational flexibility and evolvability of proteins has been reviewed by Tokuriki et al. [15], [16]. As discussed in these reviews, these studies suggest that for proteins that exist in equilibrium between a highly populated native state (interacting with a native ligand) and less populated conformers (binding to alternative partners), mutations can gradually shift the equilibrium towards a promiscuous conformer. This can eventually lead to a new dominant primary function. While mutations may be neutral with regards to the original function (i.e. hardly change the relative occupancy of the native conformer), they may cause significant increase in the occupancy of the alternative conformer. On the other hand, point mutations that reduce the occupancy of promiscuous conformers may result in a decreased flexibility (rigidification) but increased specificity (and higher affinity) for the native ligand as for example observed in the process of antibody maturation [17]. Promiscuity may therefore be a common feature of highly evolvable proteins.

Despite their highly conserved overall fold and binding sites, PDZ (PSD-95, Dlg, ZO-1) domains have been found to have surprisingly diverse binding specificities [18]. PDZ domains bind peptidic ligands, usually located at the C-terminus of partner proteins. A recent study at the genome level confirmed that this location is dominant [19], but other modes of interaction have also been reported [20]–[22].

Although a series of different classification systems have been proposed aiming to organize PDZ domains based on their preference towards peptide ligands there is no consensus on the best way of classification [23], [24] although some progress has been made towards mapping determinants of specificity [25]. PDZ specificity turned out to be unexpectedly complex as many PDZ domains are able to bind to multiple ligands that belong to different classes of peptide motifs. This property is often referred to as degenerate specificity, multivalent specificity or most commonly, binding promiscuity [10]. In addition, single peptides have been shown to bind to multiple PDZ domains. The complex picture of PDZ-peptide interactions therefore makes it rather difficult to develop a simple specificity-based classification scheme.

In addition, very little is known about what determines the specificity and promiscuity of PDZ domains. To address this question, Stiffler et al. [26] have used protein microarrays and quantitative fluorescence polarization to study the binding specificity of 157 mouse PDZ domains and found only a weak correlation between the pairwise sequence divergence of PDZ domains and their divergence in selectivity space. The fact that overall sequence similarity proved to be a poor predictor of PDZ domain function indicates that the majority of sequence variation in the PDZ family is neutral with regards to peptide-binding selectivity. This also suggests that binding specificity is mostly determined by only a subset of residues that are likely to be located in the binding pocket of the domain [26].

In order to study the sequence determinants of specific ligand recognition, Tonikian et al. [25] performed mutagenesis at ten binding site positions in the Erbin PDZ domain. As a result, they identified several mutations that altered binding specificity. Since not all of these critical residues were in direct contact with the ligand, Tonikian et al. concluded that both direct interactions and cooperative, long-range effects may play important roles in determining the specificity of PDZ domains [25].

In a recent study, using a combinatorial peptide library and site-directed mutagenesis, Shepherd et al. [27] have found that only four point mutations were enough to switch between the distinct binding specificities of the Tiam1 (T-cell lymphoma invasion and metastasis 1) PDZ and Tiam2 PDZ domains. Gee et al. [28] have come to similar conclusions after performing *in-vitro* mutagenesis studying the PDZ domains of PSD-95 (postsynaptic density protein 95) and α1-syntrophin. By identifying a few critical sequence positions, they have found that single-amino acid substitutions can alter specificity and affinity of PDZ domains for their ligands. The fact that ligand specificity relies on minor sequence modifications, while the chemistry of the binding pocket and the overall fold are well conserved, suggests a very favorable flexibility property of the PDZ domain fold [29]. PDZ domains are both versatile and robust because mutations frequently change their specificities without a loss of function [25]. Similar robustness under high mutational pressure has also been observed for other peptide-binding domains, for example the WW [30] and SH3 domains [31].

On the other hand, a number of experimental and computational studies (outlined below) have shown that the conformational dynamics of PDZ domains may also play a crucial role in determining binding specificity. These results suggest that the intrinsic fluctuations of PDZ structures are also likely to be related to the selectivity for peptide ligands. Recently, Gerek et al. [32] used a modified coarse-grained elastic network model to find characteristic residue fluctuation patterns for PDZ domains belonging to different specificity classes. By clustering these residue fluctuation profiles, they have identified common motion characteristics of Class I and Class II type PDZ domain interactions [32].

Basdevant et al. performed 20–25 ns molecular dynamics simulations of 12 PDZ domain complexes and used the MM/PBSA (Molecular Mechanics/Poisson-Boltzmann Surface Area) method to analyze electrostatic, nonpolar and configurational entropy contributions to the binding free energies [33]. Their results show that the degree to which the dynamics of the peptide ligands are coupled to those of the PDZ domains varies highly. They concluded that complex-specific dynamical or entropic responses may form the basis of the selective recognition of peptides. It is important to note that different flexible docking strategies have already been proposed to be able to incorporate the effect of binding site flexibility in structure-based drug design studies targeting PDZ domains [34], [35].

Another aspect that has been investigated is the role of temperature on binding behaviour. Staneva and Wallin [36] applied an all-atom Monte Carlo based approach to analyze various aspects of the process of peptide binding to PDZ domains. They found that the probability that peptide ligands can occupy the correct bound state in the simulations increased sharply with the decrease of temperature. In another study, Cecconi et al. [37] have analyzed the temperature-dependence of the unbinding of peptide ligands from PDZ domains. They have found that the free-energy landscape determining the kinetics of ligand escape is sensitively dependent on the temperature. However, PDZ-peptide complexes are stabilized within a physiologically relevant temperature interval.

Given all of the above, we were interested in the role of conformational dynamics in determining the ligand binding specificity of PDZ domains. In particular, given the possible relationship between flexibility and promiscuity, we wanted to examine how well the property of multi-specificity of these domains is correlated with the flexibility of their binding pockets. We were also interested to examine to what extent PDZ domains obey the conformational selection versus induced fit mechanism. We thus selected five, well-characterized, PDZ domains: Dvl2 PDZ capable of binding both C-terminal and internal (i.e. not at the terminus of a protein) peptides and shows large conformational changes between binding modes, Erbin (ERBB2 interacting protein) PDZ which binds both class I and class II ligands, but comparison with the apo structure reveals very little conformational change, InaD PDZ1 for which it is known that peptides bind in different modes, but structural information is thus far only available for one mode, PTP-BL PDZ2 for which induced fit has been predicted to be important in the binding process and GRIP1 PDZ7 for which structural studies suggest that the binding cleft is not capable of binding peptides in the expected manner for PDZ domains.

All five of the aforementioned PDZ domains are of clinical interest due to their central role in disease pathways. Four of these PDZ domains (Dvl2 PDZ, Erbin PDZ, InaD PDZ1 and PTP-BL PDZ2) are promiscuous in the sense that they are able to interact with multiple partners. However, while for example, Dvl2 PDZ is capable of interacting with peptides using different binding modes (binding both classical C-terminal and non-classical internal peptides), Erbin PDZ is able to interact only with very similar peptide binding modes.

On the basis of this, one can formulate a definition of strong promiscuity, which is the ability to interact with multiple ligands that require the binding pocket to adopt significantly different conformations. In this sense, Dvl2 PDZ is promiscuous and Erbin PDZ is not. If conformational selection plays a role in the recognition of peptides, the above-defined property of promiscuity must correlate with intrinsic conformational flexibility since the binding pocket needs to visit all different conformations required for binding multiple ligands. In this paper we explore the relationship between the dynamics, promiscuity and flexibility of PDZ domains. The results have implications for many protein-protein interaction pathways.

## Results/Discussion

**Figure 1:**
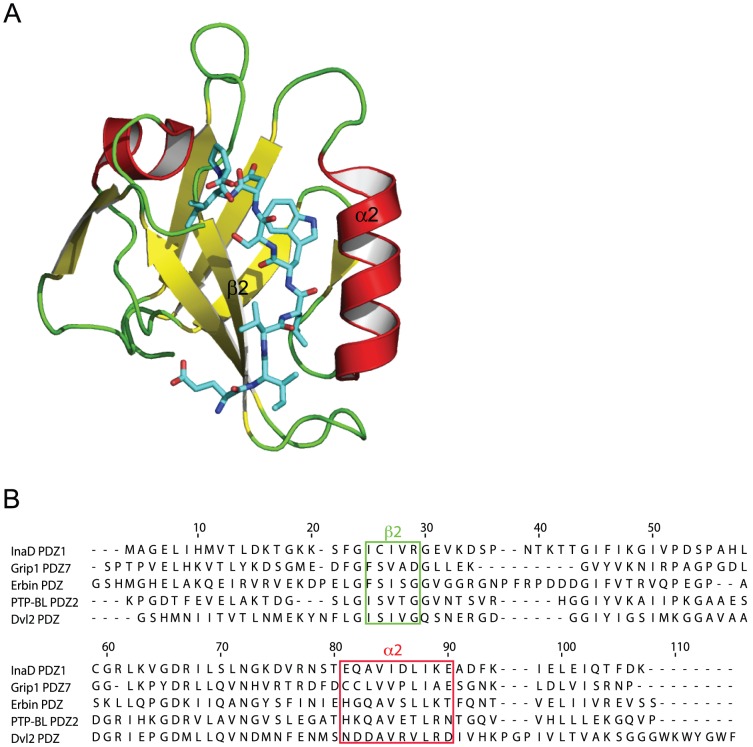
The architecture of a PDZ domain as illustrated by Dvl2 PDZ (A) shows that the peptide ligand binds (licorice representation) in between a cleft formed by a the α2-helix (except in Erbin where its called the α1) and the β2-strand. The multiple sequence alignment of the five PDZ domains used in this study are used to define the sequence regions of these two key secondary structure elements (B).

To explore the role of conformational selection and flexibility and its relationship to promiscuity of binding we examined five well-documented PDZ domains (Table 1, Figure 1) with 200 ns molecular dynamics simulations (See Methods). Although the sequence identity is between 19 and 30%, the structural similarity, as measured by the root-mean squared deviation of the Cα carbons, of these domains is high, especially in the binding site (Table 2).

**Table 1:** Summary of the 5 PDZ domains used in this study.

Name	PDB	Organism	Binding Pocket	Characteristics
Dvl2 PDZ	3cbx [22]	*Homo Sapiens*	β2: I280-G284 α2:N330-D339	Flexible, large structural variation.
Erbin PDZ	2h3l [52]	*Homo Sapiens*	β2: F334-G338 α1: H388-T397	Rigid: little structural variability.
Grip1 PDZ7	1m5z [47]	*Rattus Norvegicus*	β2: F38-D42 α2: C84-E93	Closed: unable to bind C-terminal peptides.
InaD PDZ1	1ihj [44]	*Drosophila Melanogaster*	β2: I30-R34 α2: E84-E93	Capable of different binding modes.
PTP-BL PDZ2	1gm1 [45]	*Mus musculus*	β2: I27-G31 α2: H78-N87	Induced fit binding mechanism

The table highlights the sequence regions corresponding to the α2 helix (or α1 helix in Erbin PDZ) and the β2 strand. See Methods section for details of the characteristic sequence alignment analysis used for defining binding site residues.

**Table 2:** Sequence and structural similarity of the five PDZ domains used in this study.

	Erbin PDZ	GRIP1 PDZ7	InaD PDZ1	PTP-BL PDZ2
**Dvl2 PDZ**	24.3%	19.8%	19.4%	30.1%
	1.8 Å	2.4 Å	2.0 Å	2.1 Å
	(1.1 Å)	(1.4 Å)	(1.7 Å)	(1.4 Å)
**Erbin PDZ**	-	26.4%	24.5%	25.5%
		3.5 Å	2.6 Å	3.2 Å
		(0.8 Å)	(1.0 Å)	(0.6 Å)
**GRIP1 PDZ7**	-	-	29.7%	28.6%
			1.4 Å	1.0 Å
			(1.1 Å)	(0.5 Å)
**InaD PDZ1**	-	-	-	23.4%
				1.3 Å
				(0.9 Å)

Pairwise sequence identity, RMSD based on residues belonging to secondary structural elements and RMSD between binding site residues only (values shown in brackets). Equivalent positions of the five PDZ structures are defined by their multiple sequence alignment (see Methods for details).

### Overall fluctuation measurements

To compare the inherent flexibility of the five PDZ binding pockets, we used a measure of the overall fluctuation, Θ, which reflects the mean pairwise distance variance of binding pocket residues (See Methods for details). This approach has the added advantage that it is not superposition dependent as it only depends on distances rather than coordinates. The overall fluctuation was calculated for the five conformational ensembles of the 200 ns MD simulation trajectories (40000 snapshots for each PDZ domain). We assessed the convergence of the trajectories via calculation of the root mean square inner product (RMSIP) and obtained values between 0.59 and 0.69 for the binding pocket residues (and high overlaps for the full proteins as well) from the simulations which according to Lagerge and Yonetani [38] suggests adequate convergence (see Supporting Information, Text S1, for more details). The Θ fluctuation values of the five binding pockets (i.e. the five sets of binding site residues defined by the multiple sequence alignment) are summarized in Table 3. As discussed in Methods, the Θ measure shows the size of conformational space the binding pocket explores in the simulation. Table 3 shows that despite the high structural similarity of the five binding sites (Table 2), one can see large differences in the extent of their intrinsic fluctuation. The InaD PDZ1 and Dvl2 PDZ binding sites have the most flexible binding pockets, while the binding site of Erbin PDZ is the most rigid of these five PDZ domains. The Θ value of Dvl2 PDZ is almost twice as large as that of Erbin PDZ.

**Table 3:** Overall fluctuation measure, Θ, calculated for the five PDZ binding sites based on the conformational ensembles of the 200 ns MD trajectories.

Protein	Θ_Binding pocket_
InaD PDZ1	0.077
Dvl2 PDZ	0.071
Grip1 PDZ7	0.060
PTP-BL PDZ2	0.047
Erbin PDZ	0.038

These results are in good agreements with the conclusions of experimental studies [22], [39]–[42] that have found that Erbin PDZ binding site shows little structural variability while the Dvl2 PDZ binding site is flexible showing large structural variation. The results suggest that the rigidity/flexibility of these binding sites demonstrated in other studies by comparison of apo and holo crystal structures can be explained by the intrinsic dynamics of the apo proteins.

### Comparison of Erbin to Dvl2 PDZ domains: A rigid versus flexible binding site

**Figure 2:**
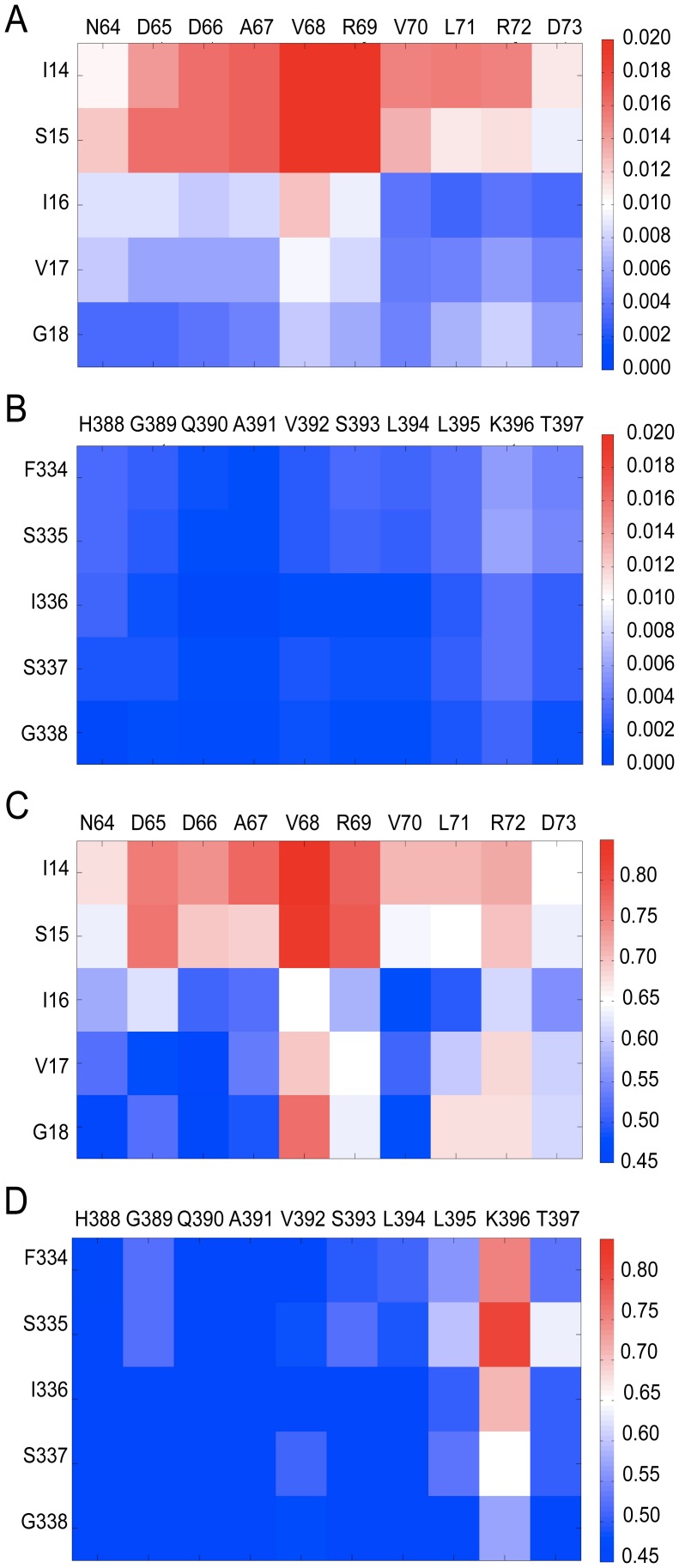
Comparison of the fluctuation patterns for the Dvl2 PDZ (A) and the Erbin PDZ (B). The Dvl2 PDZ shows considerably more fluctuation across its binding site compared to Erbin PDZ. Comparison of the flexibility patterns for Dvl2 (C) and Erbin (D). The color bar indicates the extent of fluctuation or flexibility for the pairs of residues in the matrix with red indicating the most fluctuation (or flexibility) and dark blue the least.

The flexibility of the binding pocket of the Dvl2 PDZ domain has been discussed in the literature before [22]. Therefore we decided to compare the dynamics between Dvl2 PDZ and Erbin PDZ domains. The difference in the overall fluctuation of the two binding pockets can also be seen in their fluctuation matrices (Figure 2A,B), defined as the matrix of variance of pairwise residue distances. We also define “flexibility” as a measure of the maximum range any pairwise residue distance can exhibit (see Methods). The flexibility matrices, which essentially capture extreme movements, reveal that, as expected, there are regions of high flexibility for Dvl2 PDZ. They also reveal, unexpectedly that although the fluctuation matrices suggested that Erbin PDZ is quite rigid, they also highlight that there is flexibility in terms of the distance between K396 (located at the C-terminal of the α1 helix) and the β2 strand and in particular S335 (see Supporting Information Figure S1). Taking the result of the fluctuation and flexibility matrices together suggests that a section of the binding site can open up considerably, but that these extremes in conformation are infrequent and essentially the Erbin PDZ binding site behaves as a rigid structure.

**Figure 3:**
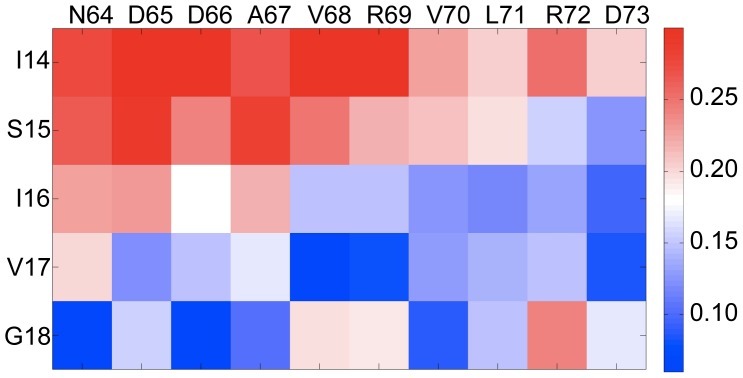
Flexibility matrix calculated for an experimental ensemble of Dvl2 PDZ structures; apo (PDB code: 2rey), and 4 ligand-bound complexes (PDB codes: 3cbx, 3cby, 3cbz, 3cc0).

To better understand the role that intrinsic dynamics might play in ligand binding to the Dvl2 PDZ domain, we performed the fluctuation and flexibility analysis on an experimentally derived ensemble. We took the structure of the apo Dvl2 PDZ domain (PDB code: 2rey) and four crystal structures of different ligand-bound conformations (PDB codes: 3cbx, 3cby, 3cbz and 3cc0 which are also referred to in the literature as the pep-C1, pep-N1, pep-N2 and pep-N3 complexes [22]). The pep-C1 structure exemplifies C-terminal ligand binding, whereas the other three illustrate internal ligand binding. The flexibility matrix was computed for this ensemble and is shown in Figure 3. The matrix shows us which binding pocket residue pairs have the largest relative displacement between the apo and ligand-bound structures. The experimentally derived flexibility matrix has remarkable similarity to the simulation-based fluctuation and flexibility pattern (Figure 2A and C) with a correlation of 0.74 and 0.68 respectively. The largest displacements seen experimentally are for residues I14 and S15 with respect to the α2 helix, which is the same as that observed in the simulations. This suggests that the Dvl2 PDZ domain is capable of visiting conformations that are consistent with the ligand-bound conformations even in the absence of ligands.

**Figure 4:**
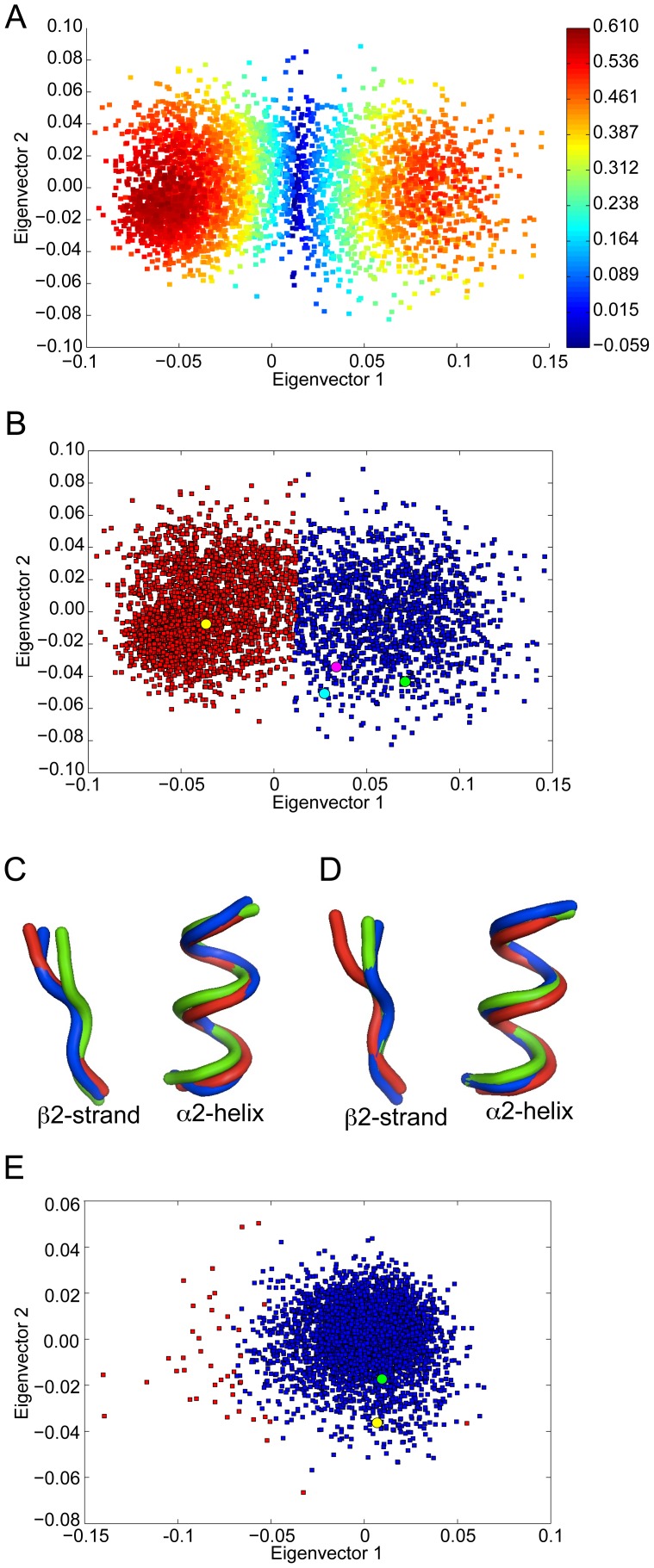
Multidimensional scaling analysis for Dvl2 PDZ colored by silhouette index values (A) and by cluster membership (B). Blue dots represent conformations that belong to cluster one; red dots those that belong to cluster two. The four crystal structures, pep-C1, pep-N1, pep-N2 and pep-N3 are represented by magenta, cyan, yellow and green dots, respectively. A comparison of the medoid conformation from cluster one to experimentally observed ligand-bound states (C) shows that medoid (blue) is closer to the conformation for pep-N3 (red) than pep-N2 (green). Conversely, the medoid conformation from cluster two (D) is closer to the conformation of pep-N2 (green) than pep-N3 (red). Multidimensional scaling analysis for the Erbin PDZ (E) reveals one major cluster (blue dots) with several outliers (red dots) defined as conformations that have dRMSD dissimilarity equal or larger than 0.8 Å from the medoid conformer.

We were interested to know how the snapshots of the MD simulations were distributed in conformational space. To that end we performed multi-dimensional scaling on snapshots taken every 50 ps (the first ns of the trajectories were excluded). The input was thus a 3981×3981 matrix containing the pairwise dRMSD dissimilarity values of 3981 conformers. Groups of similar conformers were identified with the k-means cluster analysis and clustering was validated with the silhouette index measure (see Methods). The optimal number of clusters corresponding to the maximal overall average silhouette index (*S_OVER_* = 0.411) was found to be 2. Figure 4A shows the results of multi-dimensional scaling (MDS) where conformations are represented by dots on a 2D-map and similar conformers are adjacent. The map suggests that the conformational space can be split into two distinct contiguous clusters.

When the ligand-bound structures were included in this MDS analysis (Figure 4B) it was found that one (pep-N2) resides within cluster two, whilst the other three sit within cluster one. Examination of ligand-bound conformations with, superposed on, the medoid conformations (ie. those conformations that are most representative of the ensemble) from the two clusters (Figure 4C, and D) shows that the key difference lies in the motion of I14 and S15 (at the N-terminal of the β2 strand) relative to the α2 helix. Thus the intrinsic fluctuations observed for the Dvl2 PDZ domain allow access to two distinct conformational states that have been captured in ligand-bound structures.

In contrast, MDS performed on the data for the Erbin PDZ domain shows just one cluster and the two experimental structures lie within this cluster (Figure 4E). The complex with the class I peptide is located close to the cluster center, while the complex with the class II peptide is placed closer to the edge of the cluster. The Erbin PDZ domain is promiscuous in the sense that it binds multiple peptides, but the experimental structures and this analysis shows that these peptides are essentially binding to the same conformational state and thus it does not satisfy the “strong” definition of promiscuity defined here.

### InaD PDZ1 domain has distinct conformational states

**Figure 5:**
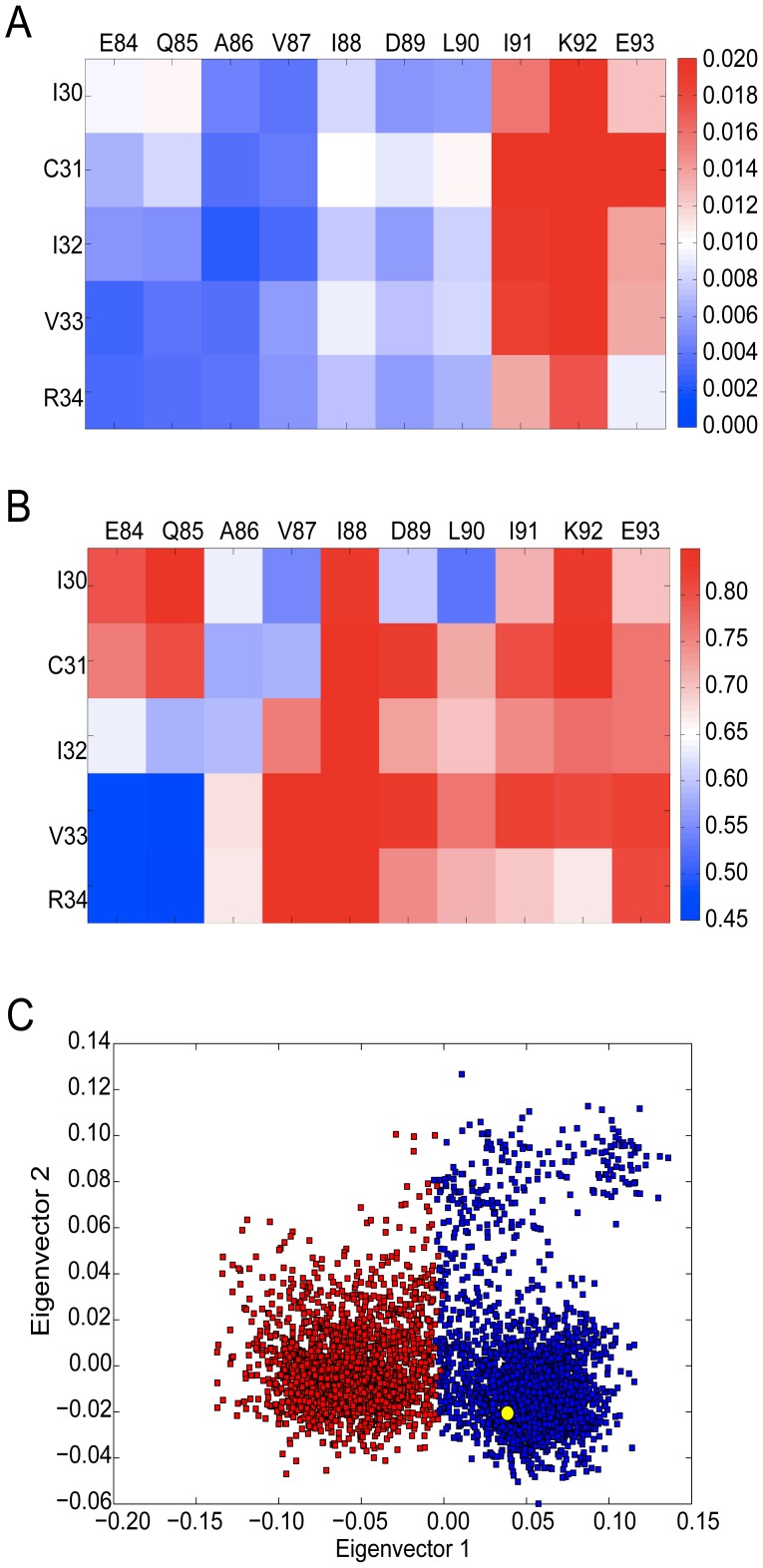
Fluctuation pattern (A) and the flexibility pattern (B) for the InaD PDZ1 domain. The MDS analysis (C) divides the data into two discrete clusters. The yellow dot is the conformation when the NorpA peptide is bound.

The binding pocket of the InaD PDZ1 domain has the largest overall fluctuation of the five PDZ binding pockets examined here (Table 3). The fluctuation matrix (Figure 5A) shows that the part of the InaD PDZ1 domain that fluctuates the most is the three residues at the C-terminal end of the α2-helix (I91, K92, and E93) with regards to the entire β2-strand. However, the flexibility matrix (Figure 5B) shows that this PDZ domain can undergo even larger distortions within the binding cleft. MDS analysis of the conformational ensemble identified two main clusters (Figure 5C) with the known experimental structure of the InaD PDZ1 domain in complex with the NorpA peptide (PDB code: 1ihj) belonging to cluster one. The overall average silhouette index, *S_OVER_* was 0.43 and as can be seen the division between clusters is not as distinct as for the Dvl2 PDZ.

The presence of two distinct clusters for InaD is intriguing and raises the question of whether the second cluster has biological relevance. Besides the NorpA peptide, the InaD PDZ1 domain has been shown to bind to the unconventional myosin NinaC. Intriguingly the experimental results [43] suggest that InaD PDZ1 may interact with NinaC in a different mode than it does with NorpA [44]. If conformational selection plays a role in this interaction, then one would expect the InaD PDZ1 domain to be relatively flexible in the apo state and able to visit distinct regions of conformational space, which is exactly what is observed here. Conformations in Cluster 1 are likely to be relevant for NorpA binding whilst excursions into Cluster 2 may be essential for NinaC peptide binding.

Taken together these data suggest that the InaD PDZ1 domain is likely to satisfy the ‘strong’ definition of promiscuity as it probably binds to different partners using considerably different binding modes. Thus we would predict from this that any structure of the InaD PDZ1-NinaC complex would be placed into Cluster 2 of the MDS analysis. These results are in support of the earlier anticipation of Kimple *et al* [44] and Wes *et al* [43] that InaD PDZ1 binds to NorpA and NinaC using different binding modes. Based on our results we would predict that the difference is expected to be in the shift of the C-terminal end of the α2-helix (I91, K92 and E93) with respect to the β2-strand.

Although InaD PDZ1 (and also Dvl2 PDZ) has distinct conformation clusters defined by k-means clustering, it is perhaps also useful to define states in terms of kinetics. We performed a temporal analysis to ascertain whether our geometrically defined states are supported by a kinetic definition simply defined by asking is the intra-cluster relaxation time faster than the inter-cluster transition time (see Supporting Information, Text S1, for details). For InaD PDZ1, the average inter-cluster transition time was 14.1 ns whilst the intra-cluster relaxation time was 100 ps. Similarly for Dvl2 PDZ, the average inter-cluster transition time was 7.03 ns whereas the intra-cluster relaxation time was again 100 ps. Thus, this analysis suggests that the conformational clusters defined by the dRMSD similarity measure correspond to kinetically separated, metastable states of the protein.

### The PTP-BL PDZ2 domain has a high induced-fit component

**Figure 6:**
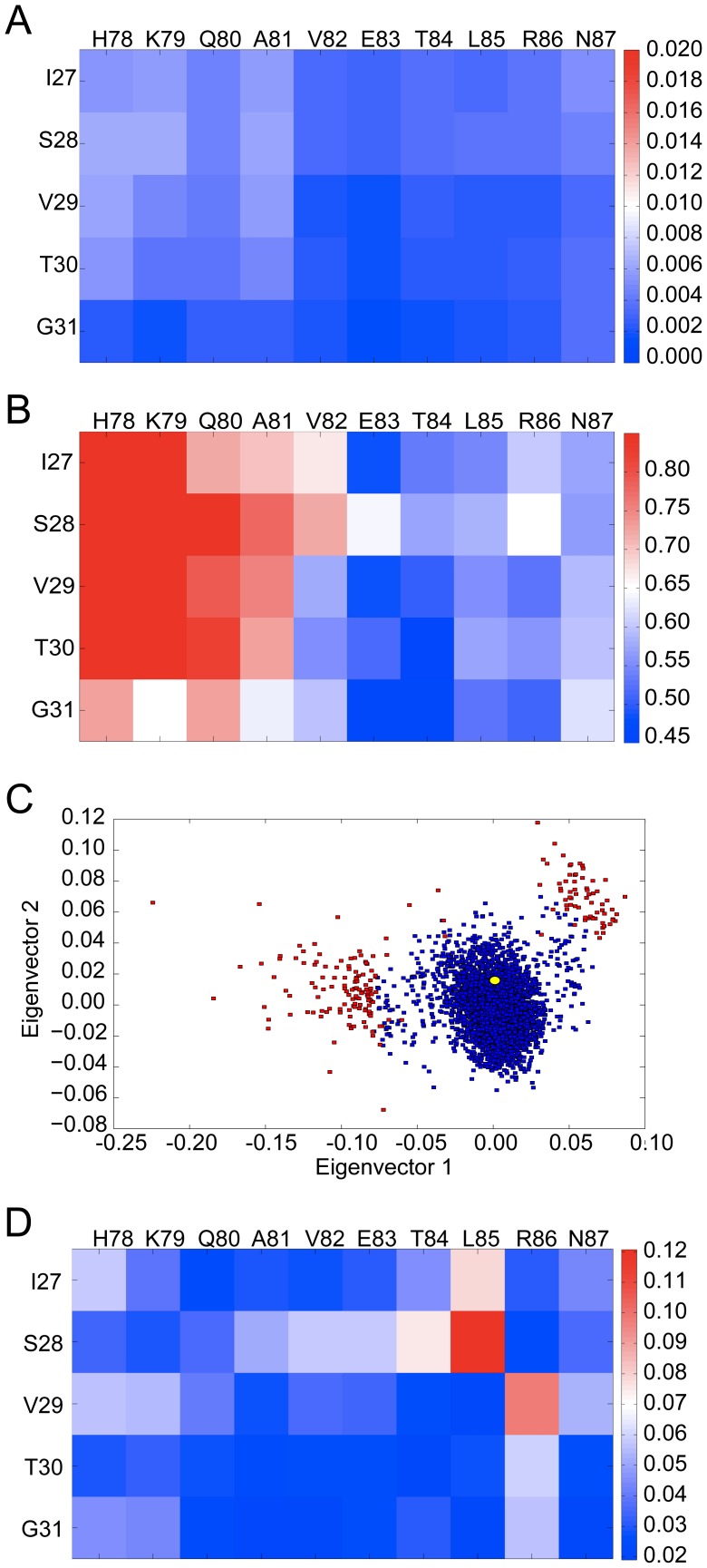
Fluctuation pattern (A) and the flexibility pattern (B) for the PTP-BL PDZ2 domain. The multidimensional scaling analysis (C) of PTP-BL PDZ2. The conformer corresponding to the APC-bound structure is shown in yellow. Outliers are indicated in red and are defined as having a dRMSD larger or equal to 0.9 Å from the medoid conformer. The mean absolute difference distance matrix (Δ) pattern (D) calculated between the APC peptide-bound conformation of PTP-BL PDZ2 and the 100 most similar MD simulation snapshots.

The fluctuation pattern of PTP-BL PDZ2 (Figure 6A) shows that this domain [45] has a considerably rigid binding site, similar to the Erbin PDZ domain. However, the flexibility pattern (Figure 6B) reveals that the N-terminal end of the α2 helix is flexible with regards to the β2 strand. MDS analysis (Figure 6C) shows that the majority of conformations appear to be distributed within a single compact cluster, which also has a large number of outliers.

The results here place the experimental ligand-bound conformation in the main conformational cluster, but that does not rule out the possibility that induced-fit plays an essential role in the binding process. Indeed for PTP-BL PDZ2, the binding to the Adenomatous Polyposis Coli-protein (APC) peptide has been proposed to occur through induced fit [46]. In order to investigate this, the structural differences between the APC-bound conformation and the most similar (neighboring) conformations sampled in the apo MD simulation were characterized using Q values (see Methods) which is introduced as a quantitative measure of similarity. Table 4 summarizes the results of this analysis for the PDZ domains where there is a complex reported. It can be seen that the complex of PTP-BL PDZ2 domain with the APC peptide is the least similar to the apo MD simulation ensemble. It has the highest Q^(1)^ and Q^(10)^ values (0.37 Å and 0.39 Å) which represent the average dRMSD dissimilarity between the ligand-bound conformer and the most similar and ten most similar stimulation snapshots, respectively. By contrast, the complex of InaD PDZ1 with the NorpA peptide has significantly lower Q^(1)^ and Q^(10)^ values (0.15 Å and 0.18 Å) indicating that this ligand-bound binding site conformation is more closely approached in these simulations. We also performed an additional simulation of the PTP-BL PDZ2 domain in complex with the APC peptide to examine whether the presence of the peptide kept conformational space closer to the ligand-bound crystal structure. As expected, the Q^(1)^ and Q^(10)^ values are lower (see Supporting Information, Text S1) for the simulation with the peptide bound compared to apo (Table 4), lending further support to the induced fit mechanism.

**Table 4:** Mean dRMSD dissimilarity between the ligand-bound conformations and the most similar, 10 most similar, 100 most similar and 200 most similar snapshots of the apo MD simulations (for details of the Q(1), Q(10), Q(100) and Q(200) measures see Methods).

Complex	Q^(1)^ (Å)	Q^(10)^ (Å)	Q^(100)^ (Å)	Q^(200)^ (Å)
Erbin PDZ: Class I peptide	0.17	0.18	0.21	0.22
Erbin PDZ: Class II peptide	0.21	0.26	0.31	0.33
Dvl PDZ: pep-C1 peptide	0.29	0.32	0.36	0.41
Dvl PDZ: pep-N1 peptide	0.30	0.36	0.44	0.48
Dvl PDZ: pep-N2 peptide	0.19	0.25	0.31	0.33
Dvl PDZ: pep-N3 peptide	0.28	0.30	0.34	0.36
InaD PDZ1: NorpA peptide	0.15	0.18	0.21	0.23
PTP-BL PDZ2: APC peptide	0.37	0.39	0.43	0.45

Although due to sampling limitations, we are unable to tell if the apo structures get any closer to the peptide-bound conformations in reality, the data presented here suggest that, out of the five PDZ domains studied here, PTP-BL PDZ2 is the most likely to involve an induced fit mechanism when binding to the APC peptide. Figure 6D shows the mean absolute difference distance matrix (Δ) pattern calculated between the peptide-bound structure and the 100 most similar snapshots. We can see that the largest deviations are found in the distances between S28 and L85 and between V29 and R86. The Δ pattern suggests that these two inter-residue distances are altered the largest extent upon binding to the APC peptide.

Visual inspection of the PTP-BL PDZ2 trajectory shows some subtle rearrangements of the protein from the starting crystal structure. The movement between residues S28 and L85 along with V29 and R86 appears to be facilitated by re-arrangement of the “pre-β2” loop and the “post-α2” loop and the movement of K10, the side-chain of which appears to act as a helix cap for the α2 helix most of the time. As these movements occur, water molecules penetrate deeper into the cleft but are then expelled as the cleft returns to conformations more similar to the starting structure. However, the whole structure appears to be further stabilized by the formation of salt-bridge between residues D22 and K50 which is not initially present in the crystal structure (see Supporting Information, Figure S2). The overall change in shape of the pocket in this extreme is similar in nature to opening of the Erbin PDZ domain (which occurs infrequently – see Supporting Information, Figure S1).

### GRIP1 PDZ7 is an example of a “closed” binding site that does not readily open

The solution structure of the GRIP1 PDZ7 domain [47] suggests that the α2/β2 binding pocket adopts a “closed conformation” and has a significantly smaller carboxyl peptide-binding site than other PDZ domains which would restrict its ability to interact with peptides. However, it is the case that other PDZ domains with similar closed pockets appear to be able to open up in order to incorporate a peptide ligand such as LARG PDZ domain [40]. Thus we examined the conformational dynamics of the GRIP1 PDZ7 domain to see if it would open up to a conformation capable of peptide binding.

**Figure 7:**
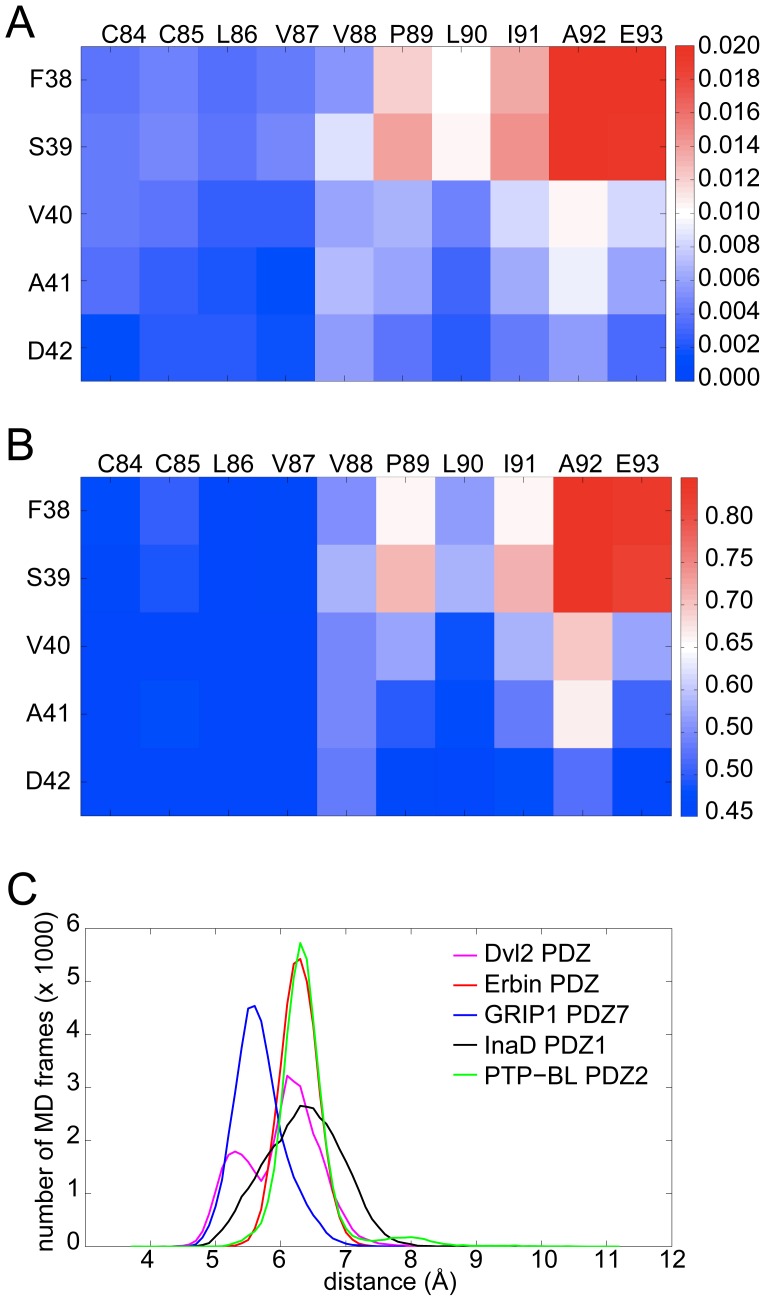
Fluctuation (A) and flexibility pattern (B) for GRIP1 PDZ7 showing that the binding site is conformationally restricted. Distribution (C) of the size of the binding cleft defined by a simple distance across the base of the cleft for equivalent residue pairs in all five PDZ domains studied here (Dvl2: R34 and E84, Erbin: G338 and H388, InaD: R34 and E84, PTP-BL: G31 and H78 and GRIP1 PDZ7: A41 and C84). Distances are taken from the MD simulations (discarding the first nanosecond).

The fluctuation and flexibility patterns of the GRIP1 PDZ7 binding pocket (Figure 7A and B) show that the N-terminal end of the β2-strand has notable fluctuation with regards to the C-terminal end of the α2-helix. On the other hand, the patterns also show that the C- terminal end of the β2-strand has little mobility with regards to the N-terminal end of the α2-helix. Since the bottom of the binding pocket is located between the C-terminal end of the β2-strand and the N-terminal end of the α2-helix, their low relative fluctuation suggests that the base of the binding site does not open significantly.

In order to examine this in more detail, and in a comparative way to the other PDZ domains studied here, the distance between the C-terminal residue of the β2-strand and the N-terminal residue of the α2-helix was used to characterize to what extent the base part of the binding pockets in all the PDZ domains is open. Figure 7C shows these distance distributions for each PDZ domain. The distributions for Erbin PDZ and PTP-BL PDZ2 are almost identical (both are approximately Gaussian functions with a mean of 6.28 Å and 6.4 Å, and standard deviation of 0.29 Å and 0.49 Å, respectively) indicating that the base parts of the binding groove of these two PDZ domains behave in a very similar fashion. The distribution of the InaD PDZ1 domain, however, has larger spread (a standard deviation of 0.58 Å), but the mean distance is about the same (6.35 Å) as for Erbin PDZ and PTP-BL PDZ2. Interestingly, the distance distribution of Dvl2 PDZ is a superposition of two Gaussian distributions (with a mean of 6.0 Å and a standard deviation of 0.58 Å). However, the location of one of the two superposed Gaussian curves agrees well with the distributions observed for Erbin PDZ and PTP-BL PDZ2.

Most importantly, the distance distribution of GRIP1 PDZ7 (which can be approximated well as a single Gaussian distribution) is significantly shifted relative to the other four distributions. It has a mean of only 5.68 Å, and a standard deviation of 0.39 Å. The probability that the base part of the binding pocket is open with an extent larger than 0.6 Å is considerably lower in the case of the GRIP1 PDZ7 domain but is high in the four other PDZ domains. These results show that the bottom of the binding groove of the GRIP1 PDZ7 domain is closed and it remains closed in the course of the 200 ns MD simulation unlike in other PDZ binding sites. This unique property of GRIP1 PDZ7 is probably the reason why this PDZ domain has been found to be unable to bind to carboxyl peptides.

### Conclusions

The intrinsic dynamics of the binding sites of five PDZ domains have been compared in this paper, based on 200 ns all-atom molecular dynamics simulations of the apo structures. Despite the remarkable structural similarity of the five PDZ folds and binding sites, their fluctuation and flexibility properties have been found to be surprisingly different. Furthermore, the differences of their mobility correlate well with differences of their functional properties suggesting that intrinsic dynamics is an important determinant of function.

The binding sites of InaD PDZ1 and Dvl2 PDZ are the most flexible of those of the five PDZ domains and this high degree of flexibility is likely to be necessary for them to be able to interact with multiple partners using significantly different binding modes, a property referred to as “strong promiscuity”. The Erbin PDZ domain, by contrast, has a rigid binding site and while it is also promiscuous, it interacts with very similar peptides using very similar binding modes. We do not count interactions with proteins at distal sites such as that reported for the Erbin-Smad3 MH2 interaction [48] which appears to be well away from the classical PDZ interaction groove. The results presented here are consistent with the proposed link between binding site flexibility and promiscuity discussed in other studies [14], [16].

Currently there is no experimental structure available of the complex of InaD PDZ1 with the NinaC peptide. Based on the results presented in this study, we predict that InaD PDZ1 interacts with NinaC in a significantly different binding mode than it does with NorpA, a conclusion also made by Kimple et al [44] and Wes et al [43]. This hypothesis should be readily testable via structural characterization experiments.

The results for PTP-BL PDZ2 have revealed that the conformational space explored by the apo protein is the most different from the APC peptide-bound conformation compared to the other PDZ-peptide complexes. These results, in accordance with experimental data, suggest that the induced fit mechanism may be crucially involved in the binding of PTP-BL PDZ2 to the APC peptide and play a larger role in the recognition mechanism compared to other PDZ domains. Overall it seems likely that conformational selection and induced fit both appear to play roles in binding of PDZ domains to their peptides. One can formulate the two mechanisms into distinct roles; Firstly, conformational selection seems to be an essential mechanism for PDZ domains to visit regions of the conformational space that are close to different ligand-bound states. Visiting these regions is probably necessary for the formation of weak (initial) complexes. Once a weak complex is formed, the induced fit mechanism, as a fine-tuning step, could lead to minor changes in the shape of the binding pocket stabilizing the PDZ-peptide complex. The extent to which these mechanisms are required is likely variable across the PDZ domain family.

The MD simulations confirm that GRIP1 PDZ7 has a closed canonical binding site which is consequently unable to accommodate carboxyl peptides. The binding pocket does not appear to undergo a transition from its closed state to an open state in the course of the 200 ns trajectory. These results agree with the experimental observations that GRIP1 PDZ7 cannot interact with carboxyl ligands.

The results highlight how one fold can exhibit quite different dynamics. For PDZ domains this issue should be borne in mind when considering structure-based drug-design [49]. Considering conformational selection in the docking strategies of virtual screening is a promising new paradigm recently reviewed by Amaro and Li [50]. Furthermore, describing binding site flexibility was suggested to be crucial for designing compounds of high selectivity for a given drug target [51]. As the dynamics of the PDZ binding pocket seems to be a key factor determining the ability to interact with different peptides, the flexibility of the binding site should also be taken into account alongside steric and electrostatic effects [52] in rational drug design. From this work we would anticipate that intrinsic dynamics would play a role in other systems ranging from influencing large domain movements through to allosteric transitions. As simulation times approach experimental timescale, particularly for NMR, it will become possible to assess how well these observations fit into solvable models for conformational selection and induced fit such as the one proposed by Zhou [53].

## Methods

### Molecular Dynamics simulations

All-atom 200 ns MD simulations were performed for the five apo PDZ domains summarized in Table 1 with the GROMACS software package [54], [55] using the OPLS force field [56] in an NPT ensemble. Pressure coupling was performed using the Berendsen barostat with a time constant, tau, of 1.0 ps. The systems were coupled using a Berendsen heat bath [57] with a tau value of 0.1 ps. Electrostatics were treated with a Particle Mesh Ewald scheme with a real-space cut-off of 10 Å. The neighbour list cut-off was also set to 10 Å and was updated every 10 steps. The proteins were solvated in explicit SPC water [58] and Na^+^ and Cl^−^ ions added to make up a neutral solution of 150 mM. A short steepest descents minimization of 225000 steps was performed, followed by a short restrained run of 200 ps whereby the Cα atoms of the protein were restrained by a harmonic potential with a force constant of 1000 kJmol^−1^ mn^−2^. Snapshots from the trajectories were saved every 5 ps for analysis. Convergence was assessed via root mean square inner product (RMSIP) between sections of the trajectories (see Supporting Information, Text S1, for more details).

### Measures of structural similarity

Let A and B denote two proteins that consist of *N_A_* and *N_B_* residues, respectively. In this study, residues are represented by their α-carbon atoms. An alignment between the two structures defines a mapping between the two sets of residues. Let *N* denote the number of aligned residue pairs (after removing positions aligned to gaps). The two sets of aligned residues are described by the *NxN* distance matrices of their α-carbon atoms denoted by *d^A^* and *d^B^*: i.e. the matrix entry 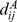 is the distance of α-carbon atoms of aligned residues *i* and *j* in structure *A*.

### Difference distance matrix

The difference distance matrix δ between structure A and B is defined as:
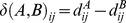
(1)


Positive entries in this matrix indicate pairs of atoms of larger distance in structure *A* than in structure *B*. This matrix can be used to characterize the location and extent of structural differences between two different proteins or two conformations of the same protein.

### dRMSD dissimilarity

The dRMSD (distance root mean square deviation) measure of dissimilarity between the two structures is defined as:
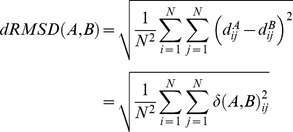
(2)


We use this measure instead of the standard RMSD dissimilarity because dRMSD is not dependent on structural superposition.

### Characterizing conformational dynamics

Let *S = {S_1_, S_2_, …, S_K_}* denote an ensemble of conformations of a protein represented by its α-carbon atoms. Let the number of its residues be *N*.

### Fluctuation matrix

We define an *NxN* matrix as the *F* fluctuation matrix, which describes the extent of the pairwise fluctuation of α-carbon atoms. Matrix *F* contains the variances of the distance of each α-carbon pair, where the variance is calculated over the whole ensemble. It is precisely defined as: 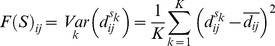
(3)where 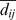 is the mean distance of α-carbon atoms *i* and *j* in the ensemble. We have previously described the use of a similar matrix where standard deviation rather than variance of the distances was used [59].

### Flexibility matrix

Although variance describes the spread of a distance distribution characterizing the relative fluctuation of two atoms, it is not always informative about how much the distance between two atoms can change. Even if the distance of two atoms significantly deviates from their mean distance in some conformations, the variance may still be low provided that most of the variation is around the mean. To measure the pairwise flexibility of two atoms (i.e. the maximal difference of their distance in the ensemble), the flexibility matrix denoted as *X* is introduced. Matrix *X* describes the range of distance distribution for each pair of atoms:
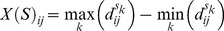
(4)


Note that the above definitions of *F* and *X* matrices allow that two pairs of atoms that have equal pairwise fluctuation can have considerably different pairwise flexibility.

### Overall fluctuation

While the *F* matrix contains pairwise atomic fluctuation values, a measure of the overall fluctuation of the whole structure (or a subset of residues) was also introduced. This overall fluctuation measure denoted by Θ was defined as the root mean square of dRMSD dissimilarity of each structure with regards the mean distance matrix calculated for the whole ***S*** ensemble. In other words, Θ is a measure for the size of conformational space the protein explores in the ensemble. It is easy to see that the above definition is equivalent to the root mean of the entries of *F* fluctuation matrix calculated for the same conformational ensemble. The precise definition of overall fluctuation is therefore
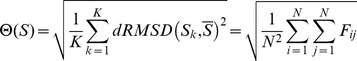
(5)where 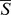 is the mean distance matrix of the ensemble.

### Binding site residues

Equivalent binding site residues were defined on the basis of a multiple sequence alignment (MSA) (Figure 1B). The binding groove of PDZ domains is located between the β2 strand and the α2 helix. Two sequence regions were therefore selected in the MSA that correspond to the conserved structural elements of the β2 strand and α2 helix (or α1 helix, in Erbin PDZ). The binding sites were characterized by 5×10 submatrices of the δ, F and X matrices describing the relative structural difference, fluctuation and flexibility of the α-helix and the β-strand.

### k-means clustering

MD simulation trajectory snapshots were clustered with k-mean cluster analysis, a simple unsupervised learning algorithm [60], [61]. The method can be used for partitioning *N* data points (here, protein conformations) into *k* disjoint subsets (or clusters) denoted by *C_1_ , C_2_ , …, C_k_* . The parameter *k* is fixed a priori. The goal of the algorithm is to find the optimal partitioning of conformations to minimize the within-cluster sum of squares (WCSS):
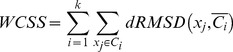
(6)where the dRMSD measure is used to capture the similarity of conformations and *C_i_* is the mean distance matrix of cluster *i*. Since *k* is an arbitrary parameter, the goodness of clustering results was estimated using the Silhouette Index cluster validity measure (see below) [62]. The optimal *k*-value that provided the highest overall average Silhouette Index was selected.

### Silhouette Index

Once the conformational ensemble is clustered, the following Silhouette Index measure is calculated for each conformation:
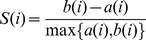
(7)where *a(i)* is the average dRMSD dissimilarity of conformation *i* to all other conformations in the same cluster and *b(i)* is the minimum of average dRMSD dissimilarities of conformation *i* to all other clusters. The silhouette index is between −1 and 1: if *S(i)* it is close to 1, it means, the conformation is well-clustered; if *S(i)* is close to 0, it means the conformation could be assigned to another cluster as well; if *S(i)* is close to −1, it means the conformation is misclassified. The goodness of clustering result was then measured by the overall average silhouette index *S_OVER_* which is simply the average of *S(i)* for all conformations in the ensemble:
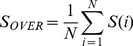
(8)


### Classical multidimensional scaling

Multidimensional scaling (MDS) (also known as Principal Coordinates Analysis) is a dimensionality reduction method often used to visualize high-dimensional data on a two-dimensional map [63]. The input of the method is a dissimilarity matrix that contains distances (dissimilarities) between pairs of objects calculated in a high-dimensional space. The output is a configuration of points embedded into lower (ideally, two or three)-dimensions. In Classical MDS (CMDS) (also referred to as Torgerson-Gower scaling) [64] used in this study, the goal is that the Euclidean distances between the outputted points should approximately reproduce the original dissimilarity matrix.

### Neighboring conformers

In order to study the difference between induced fit and conformational selection binding, a simple definition is introduced to measure how similar conformations are sampled in an apo simulation to a given experimental ligand-bound structure. Let *S^(k)^* denote the set of *k* most similar conformations (neighboring conformers) with regards to a reference experimental structure *E* (ranked based on the dRMSD dissimilarity measure). The following *Q^(k)^* value is defined as the average dRMSD dissimilarity of conformations in *S^(k)^* with regards to structure *E*:
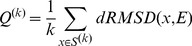
(9)


In this study the quantities *Q^(1)^, Q^(10)^, Q^(100)^ and Q^(200)^* were used to characterize the similarity of the most similar, 10 most similar, 100 most similar and 200 most similar conformations to an experimental ligand-bound structure of interest.

## Supporting Information

Figure S1 (A) Time series plot of the S335-K396 Cα-Cα distance in the Erbin PDZ domain during the 200 ns simulation. These two residues are located at the N-terminal end of β2-strand and C-terminal end of α2-helix, respectively, therefore their distance represents the size of the top part of the binding pocket. Those frames at which the distance is larger than 14 Å are highlighted in red. The ratio of frames where the distance is greater than 14 Å to the total number of frames is only 0.009 (i.e. 359 frames). For comparison, the same ratio calculated for the corresponding distance in the InaD PDZ1 domain is 0.5514 (i.e. 21947 frames). In other words, as discussed in the manuscript, the opening of the top part of the binding site is infrequent in the Erbin PDZ domain compared to for example InaD PDZ1. (B) Cartoon of the frame where this distance is maximal (17.3 Å). The opening of the binding cleft is clearly observed by the separation between I336 and V392. (C) Cartoon of the medoid frame for comparison where the distance is 11.5 Å.
(PDF)

Figure S2 (A) Shows the starting conformation with some notable observations: i) K20 acts as a helix cap to the α2 helix ii) H78 makes a hydrogen bond across the cleft to the backbone oxygen of V29 iii) K50 and D22 are not in close proximity. (B) a snapshot taken at 20.4 ns when the distance between the Cα of V29 and L85 is at its maximum. Prior to this, the loops preceding the β2 strand and following the α2 helix exhibit movements that result in the K20 helix cap moving away and allowing water to penetrate further into the cleft between V29 and L85. The H78-V29 hydrogen bond is also broken. (C) The cleft returns to a conformation similar to the starting structure, but the reformation has also allowed the formation of a salt-bridge between K50 and D22 which seems to exert a stabilizing effect on the fold.
(PDF)

Text S1 Text file containing details of the significance analysis, assessment of convergence, kinetic analysis of conformational states and a description of the holo simulation for PTP-BL PDZ2.
(PDF)

## References

[1] Calderone V, Folli C, Marchesani A, Berni R, Zanotti G (2002) Identification and structural analysis of a zebrafish apo and holo cellular retinol-binding protein. J Mol Biol 321: 527–535. 12162964 10.1016/s0022-2836(02)00628-9

[2] Done SH, Brannigan JA, Moody PC, Hubbard RE (1998) Ligand -induced conformational change in penicillin acylase. J Mol Biol 284: 463–475. 9813130 10.1006/jmbi.1998.2180

[3] Takeda M, Ogino S, Umemoto R, Sakakura M, Kajiwara M, et al (2006) Ligand-induced structural changes of the CD44 hyaluronan-binding domain revealed by NMR. J Biol Chem 281: 40089–40095. 17085435 10.1074/jbc.M608425200

[4] Boehr DD, Nussinov R, Wright PE (2009) The role of dynamic conformational ensembles in biomolecular recognition. Nat Chem Biol 5: 789–796. 19841628 10.1038/nchembio.232 PMC2916928

[5] Csermely P, Palotai R, Nussinov R (2010) Induced fit, conformational selection and independent dynamic segments: an extended view of binding events. Trends Biochem Sci 35: 539–546. 20541943 10.1016/j.tibs.2010.04.009 PMC3018770

[6] Silva D-A, Bowman GR, Sosa-Peinado A, Huang X (2011) A role for both conformational selection and induced fit in ligand binding by the LAO protein. PLoS Comput Biol 7: e1002054. 21637799 10.1371/journal.pcbi.1002054 PMC3102756

[7] Tobi D, Bahar I (2005) Structural changes involved in protein binding correlate with intrinsic motions of proteins in the unbound state. Proc Natl Acad Sci U S A 102: 18908–18913. 16354836 10.1073/pnas.0507603102 PMC1323175

[8] Wlodarski T, Zagrovic B (2009) Conformational selection and induced fit mechanism underlie specificity in noncovalent interactions with ubiquitin. Proc Natl Acad Sci U S A 106: 19346–19351. 19887638 10.1073/pnas.0906966106 PMC2780739

[9] Hammes GG, Chang Y-C, Oas TG (2009) Conformational selection or induced fit: A flux description of reaction mechanism. Proc Nat Acad Sci USA 106: 13737–13741. 19666553 10.1073/pnas.0907195106 PMC2728963

[10] Schreiber G, Keating AE (2011) Protein binding specificity versus promiscuity. Curr Opin Struc Biol 21: 50–61. 10.1016/j.sbi.2010.10.002 PMC3053118 21071205

[11] James LC, Roversi P, Tawfik DS (2003) Antibody multispecificity mediated by conformational diversity. Science 299: 1362–1367. 12610298 10.1126/science.1079731

[12] Lange OF, Lakomek N-A, Farès C, Schröder GF, Walter KFA, et al (2008) Recognition dynamics up to microseconds revealed from an RDC-derived ubiquitin ensemble in solution. Science 320: 1471–1475. 18556554 10.1126/science.1157092

[13] Muralidhara BK, Sun L, Negi S, Halpert JR (2008) Thermodynamic Fidelity of the Mammalian Cytochrome P450 2B4 Active Site in Binding Substrates and Inhibitors. J Mol Biol 377: 232–245. 18241887 10.1016/j.jmb.2007.12.068

[14] Skopalik J, Anzenbacher P, Otyepka M (2008) Flexibility of human cytochromes P450: Molecular dynamics reveals differences between CYPs 3A4, 2C9, and 2A6, which correlate with their substrate preferences. J Phys Chem 112: 8165–8173. 10.1021/jp800311c 18598011

[15] Tokuriki N, Tawfik DS (2009) Stability effects of mutations and protein evolvability. Curr Opin Struc Biol 19: 596–604. 10.1016/j.sbi.2009.08.003 19765975

[16] Tokuriki N, Tawfik DS (2009) Protein Dynamism and Evolvability. Science 324: 203–207. 19359577 10.1126/science.1169375

[17] Thorpe IF, Brooks CL (2007) Molecular evolution of affinity and flexibility in the immune system. Proc Natl Acad Sci U S A 104: 8821–8826. 17488816 10.1073/pnas.0610064104 PMC1885586

[18] Sheng M, Sala C (2001) PDZ domains and the organization of supramolecular complexes. Ann Rev Neurosci 24: 1–29. 11283303 10.1146/annurev.neuro.24.1.1

[19] Chimura T, Launey T, Ito M Evolutionarily conserved bias of amino-acid usage refines the definition of PDZ-binding motif. BMC Genomics 12: 300. 21649932 10.1186/1471-2164-12-300 PMC3138430

[20] Brenman JE, Chao DS, Gee SH, McGee AW, Craven SE, et al (1996) Interaction of nitric oxide synthase with the postsynaptic density protein PSD-95 and alpha1-syntrophin mediated by PDZ domains. Cell 84: 757–767. 8625413 10.1016/s0092-8674(00)81053-3

[21] Hillier BJ, Christopherson KS, Prehoda KE, Bredt DS, Lim WA (1999) Unexpected modes of PDZ domain scaffolding revealed by structure of nNOS-syntrophin complex. Science 284: 812–815. 10221915

[22] Zhang Y, Appleton BA, Wiesmann C, Lau T, Costa M, et al (2009) Inhibition of Wnt signaling by Dishevelled PDZ peptides. Nat Chem Biol 5: 217–219. 19252499 10.1038/nchembio.152

[23] Bezprozvanny I, Maximov A (2002) PDZ domains: evolving classification. FEBS Letts 512: 347–349.

[24] Vaccaro P, Dente L (2002) PDZ domains: troubles in classification. FEBS Letts 512: 345–346. 11852108 10.1016/s0014-5793(02)02220-2

[25] Tonikian R, Zhang Y, Sazinsky SL, Currell B, Yeh J-H, et al (2008) A specificity map for the PDZ domain family. PLoS Biol 6: e239. 18828675 10.1371/journal.pbio.0060239 PMC2553845

[26] Stiffler MA, Chen JR, Grantcharova VP, Lei Y, Fuchs D, et al (2007) PDZ domain binding selectivity is optimized across the mouse proteome. Science 317: 364–369. 17641200 10.1126/science.1144592 PMC2674608

[27] Shepherd TR, Hard RL, Murray AM, Pei D, Fuentes EJ (2011) Distinct ligand specificity of the Tiam1 and Tiam2 PDZ domains. Biochemistry 50: 1296–1308. 21192692 10.1021/bi1013613 PMC3059893

[28] Gee SH, Quenneville S, Lombardo CR, Chabot J (2000) Single-amino acid substitutions alter the specificity and affinity of PDZ domains for their ligands. Biochemistry 39: 14638–14646. 11087420 10.1021/bi001633t

[29] De Los Rios P, Cecconi F, Pretre A, Dietler G, Michielin O, et al (2005) Functional dynamics of PDZ binding domains: A normal-mode analysis. Biophys J 89: 14–21. 15821164 10.1529/biophysj.104.055004 PMC1366512

[30] Russ WP, Lowery DM, Mishra P, Yaffe MB, Ranganathan R (2005) Natural-like function in artificial WW domains. Nature 437: 579–583. 16177795 10.1038/nature03990

[31] Panni S, Dente L, Cesareni G (2002) In vitro evolution of recognition specificity mediated by SH3 domains reveals target recognition rules. J Biol Chem 277: 21666–21674. 11929862 10.1074/jbc.M109788200

[32] Gerek ZN, Ozlem K, Ozkan SB (2009) Identification of specificity and promiscuity of PDZ domain interactions through their dynamic behavior. Proteins 77: 796–811. 19585657 10.1002/prot.22492

[33] Basdevant N, Weinstein H, Ceruso M (2006) Thermodynamic basis for promiscuity and selectivity in protein-protein interactions: PDZ domains, a case study. J Am Chem Soc 128: 12766–12777. 17002371 10.1021/ja060830y PMC2570209

[34] Gerek ZN, Ozkan SB (2010) A flexible docking scheme to explore the binding selectivity of PDZ domains. Prot Sci 19: 914–928. 10.1002/pro.366 PMC2868235 20196074

[35] Niv MY, Weinstein H (2005) A flexible docking procedure for the exploration of peptide binding selectivity to known structures and homology models of PDZ domains. J Am Chem Soc 127: 14072–14079. 16201829 10.1021/ja054195s

[36] Staneva I, Wallin S (2011) Binding free energy landscape of domain-peptide interactions. PLoS Comput Biol 7: e1002131. 21876662 10.1371/journal.pcbi.1002131 PMC3158039

[37] Cecconi F, De Los Rios P, Piazza F (2007) Diffusion-limited unbinding of small peptides from PDZ domains. J Phys Chem B 111: 11057–11063. 17725342 10.1021/jp0730390

[38] Laberge M, Yonetani T (2008) Molecular dynamics simulations of hemoglobin A in different states and bound to DPG: Effector-linked perturbation of tertiary conformations and HbA concerted dynamics. Biophys J 94: 2737–2751. 18096633 10.1529/biophysj.107.114942 PMC2267116

[39] Ernst A, Sazinsky SL, Hui S, Currell B, Dharsee M, et al (2009) Rapid evolution of functional complexity in a domain family. Sci Sig 2: ra50. 10.1126/scisignal.2000416 19738200

[40] Liu J, Zhang J, Yang Y, Huang H, Shen W, et al (2008) Conformational change upon ligand binding and dynamics of the PDZ domain from leukemia-associated Rho guanine nucleotide exchange factor. Prot Sci 17: 1003–1014. 10.1110/ps.073416508 PMC2386735 18411422

[41] Shan J, Shi D-L, Wang J, Zheng J (2005) Identification of a specific inhibitor of the Dishevelled PDZ domain. Biochemistry 44: 15495–15503. 16300398 10.1021/bi0512602

[42] Shan J, Zheng J (2009) Optimizing Dvl PDZ domain inhibitor by exploring chemical space. J Comp-aid Mol Des 23: 37–47. 10.1007/s10822-008-9236-1 PMC2648812 18780146

[43] Wes PD, Xu X-ZS, Li H-S, Chien F, Doberstein SK, et al (1999) Termination of phototransduction requires binding of the NINAC myosin III and the PDZ protein INAD. Nat Neurosci 2: 447–453. 10321249 10.1038/8116

[44] Kimple ME, Siderovski DP, Sondek J (2001) Functional relevance of the disulfide-linked complex of the N-terminal PDZ domain of InaD with NorpA. EMBO J 20: 4414–4422. 11500369 10.1093/emboj/20.16.4414 PMC125561

[45] Walma T, Spronk CAEM, Tessari M, Aelen J, Schepens J, et al (2002) Structure, dynamics and binding characteristics of the second PDZ domain of PTP-BL. J Mol Biol 316: 1101–1110. 11884147 10.1006/jmbi.2002.5402

[46] Gianni S, Walma T, Arcovito A, Calosci N, Bellelli A, et al (2006) Demonstration of long-range interactions in a PDZ domain by NMR, kinetics, and protein engineering. Structure 14: 1801–1809. 17161370 10.1016/j.str.2006.10.010

[47] Feng W, Fan J-S, Jiang M, Shi Y-W, Zhang M (2002) PDZ7 of glutamate receptor interacting protein binds to its target via a novel hydrophobic surface area. J Biol Chem 277: 41140–41146. 12196542 10.1074/jbc.M207206200

[48] Déliot N, Chavent M, Nourry C, Lécine P, Arnaud C, et al (2009) Biochemical studies and molecular dynamics simulations of Smad3-Erbin interaction identify a non-classical Erbin PDZ binding. Biochem Biophys Res Commun 378: 360–365. 19013433 10.1016/j.bbrc.2008.10.175

[49] Bolia A, Gerek ZN, Keskin O, Banu Ozkan S, Dev KK (2012) The binding affinities of proteins interacting with the PDZ domain of PICK1. Proteins 80: 1393–408. 22275068 10.1002/prot.24034

[50] Amaro RE, Li WW (2010) Emerging methods for ensemble-based virtual screening. Curr Top Med Chem 10: 3–13. 19929833 10.2174/156802610790232279 PMC3086266

[51] Huggins DJ, Sherman W, Tidor B (2012) Rational approaches to improving selectivity in drug design. J Med Chem 55: 1424–1444. 22239221 10.1021/jm2010332 PMC3285144

[52] Appleton BA, Zhang Y, Wu P, Yin JP, Hunziker W, et al (2006) Comparative structural analysis of the Erbin PDZ domain and the first PDZ domain of ZO-1. J Biol Chem 281: 22312–22320. 16737969 10.1074/jbc.M602901200

[53] Zhou H-X (2010) From induced fit to conformational selection: A continuum of binding mechanism controlled by the timescale of conformational transitions. Biophys J 98: L15–L17. 20303846 10.1016/j.bpj.2009.11.029 PMC2849054

[54] Hess B, Kutzner C, van der Spoel D, Lindahl E (2008) GROMACS 4: Algorithms for highly efficient, load-balanced, and scalable molecular simuation. J Chem Theory Comput 4: 435–447. 26620784 10.1021/ct700301q

[55] van der Spoel D, Lindahl E, Hess B, Groenhof G, Mark AE, et al (2005) GROMACS: fast, flexible and free. J Comput Chem 26: 1701–1718. 16211538 10.1002/jcc.20291

[56] Jorgensen WL, Maxwell DS, Tirado-Rives J (1996) Development and testing of the OPLS all-atom force field on conformational energetics and properties of organic liquids. J Am Chem Soc 118: 11225–11236.

[57] Berendsen HJC, Postma JPM, van Gunsteren WF, DiNola A, Haak JR (1984) Molecular dynamics with coupling to an external bath. J Chem Phys 81: 3684–3690.

[58] Berendsen HJC, Postma JPM, van Gunsteren WF, Hermans J (1981) Intermolecular Forces. Dordrecht: Reidel.

[59] Münz M, Lyngsø R, Hein J, Biggin PC (2010) Dynamics-based alignment of proteins: An alternative approach to quantify dynamic similarity. BMC Bioinformatics 11: 118. 20398246 10.1186/1471-2105-11-188 PMC2868010

[60] MacQueen JB. (1967) Some methods for classification and analysis of multivariate observations. University of California Press. pp. 281–297.

[61] Hartigan JA, Wong MA (1979) A K-means clustering algorithm. J Roy Stat Soc 28: 100–108.

[62] Rousseeuw PJ (1987) Silhouettes: a graphical aid to the interpretation and validation of cluster analysis. Comp Appl Mathematics 20: 53–65.

[63] Borg I, Groenen PJF (2005) Modern multidimensional scaling. New York: Springer.

[64] Torgerson WS (1952) Multidimensional scaling, I: theory and method. Psychimetrika 17: 401–419.

