# Supplementary figures and images for: The Role of Flexibility and Conformational Selection in the Binding Promiscuity of PDZ Domains
Source: PLoS Comput Biol. 2012 Nov 1;8(11):e1002749. doi: 10.1371/journal.pcbi.1002749 (PMC3486844; doi:10.1371/journal.pcbi.1002749)

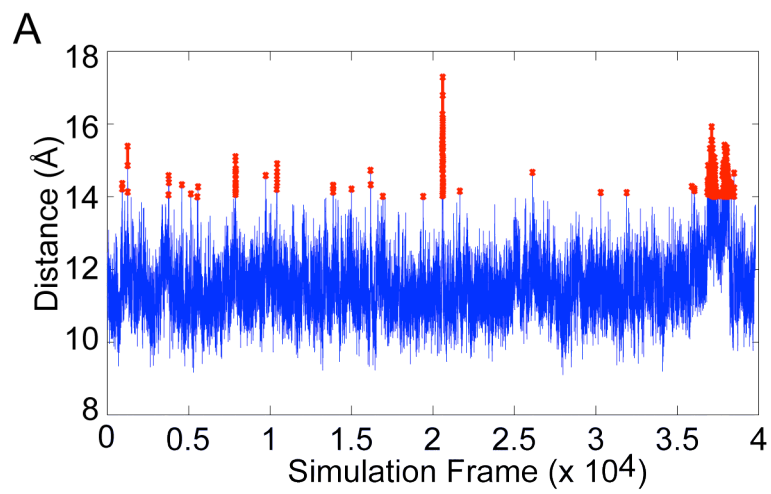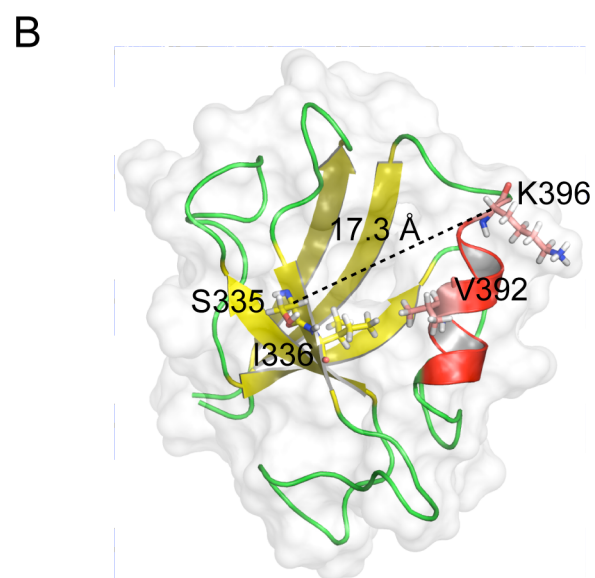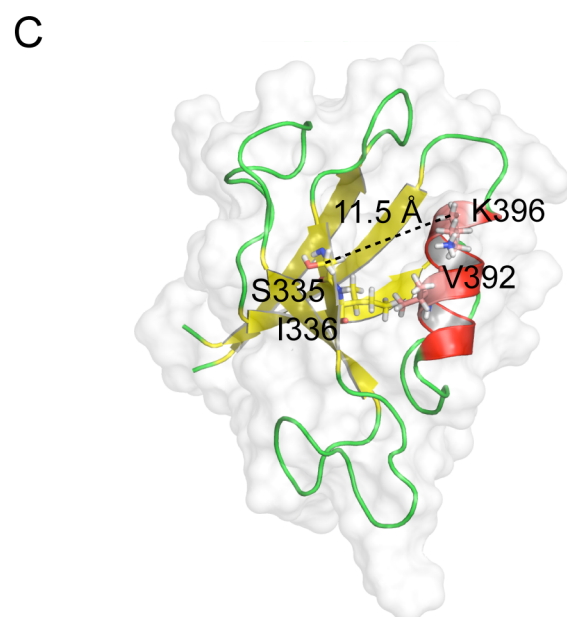

Supplement: Figure S1 — (A) Time series plot of the S335-K396 Cα-Cα distance in the Erbin PDZ domain during the 200 ns simulation. These two residues are located at the N-terminal end of β2-strand and C-terminal end of α2-helix, respectively, therefore their distance represents the size of the top part of the binding pocket. Those frames at which the distance is larger than 14 Å are highlighted in red. The ratio of frames where the distance is greater than 14 Å to the total number of frames is only 0.009 (i.e. 359 frames). For comparison, the same ratio calculated for the corresponding distance in the InaD PDZ1 domain is 0.5514 (i.e. 21947 frames). In other words, as discussed in the manuscript, the opening of the top part of the binding site is infrequent in the Erbin PDZ domain compared to for example InaD PDZ1. (B) Cartoon of the frame where this distance is maximal (17.3 Å). The opening of the binding cleft is clearly observed by the separation between I336 and V392. (C) Cartoon of the medoid frame for comparison where the distance is 11.5 Å. (PDF) [file pcbi.1002749.s001.pdf]

A

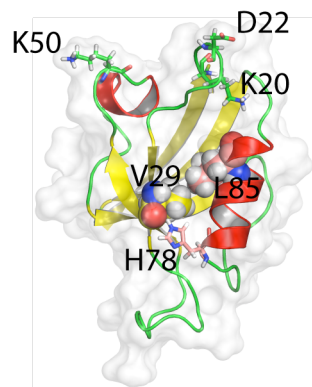

B

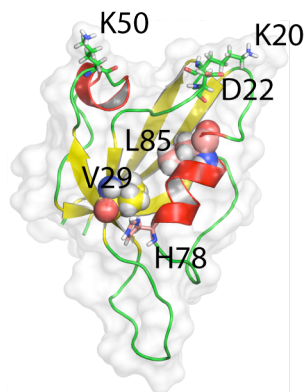

C

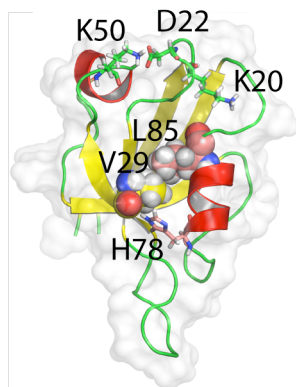

Supplement: Figure S2 — (A) Shows the starting conformation with some notable observations: i) K20 acts as a helix cap to the α2 helix ii) H78 makes a hydrogen bond across the cleft to the backbone oxygen of V29 iii) K50 and D22 are not in close proximity. (B) a snapshot taken at 20.4 ns when the distance between the Cα of V29 and L85 is at its maximum. Prior to this, the loops preceding the β2 strand and following the α2 helix exhibit movements that result in the K20 helix cap moving away and allowing water to penetrate further into the cleft between V29 and L85. The H78-V29 hydrogen bond is also broken. (C) The cleft returns to a conformation similar to the starting structure, but the reformation has also allowed the formation of a salt-bridge between K50 and D22 which seems to exert a stabilizing effect on the fold. (PDF) [file pcbi.1002749.s002.pdf]
