# Supplementary material for: The Role of Flexibility and Conformational Selection in the Binding Promiscuity of PDZ Domains
Source: PLoS Comput Biol. 2012 Nov 1;8(11):e1002749. doi: 10.1371/journal.pcbi.1002749 (PMC3486844; doi:10.1371/journal.pcbi.1002749)
Supplement: Text S1 — Text file containing details of the significance analysis, assessment of convergence, kinetic analysis of conformational states and a description of the holo simulation for PTP-BL PDZ2. (PDF) [file pcbi.1002749.s003.pdf]

Supporting Information Text S1 for Münz et al.

Significance analysis

In this paper we use a distance-based metric to assess the protein fluctuation (see for example Table 3 of the main manuscript). As the magnitudes of these measurements tend to be a lot smaller than the more familiar root mean squared deviation (RMSD) or root means squared fluctuation (RMSF) we provide here a statistical framework with which to support our statements of significance in the main manuscript. To this end, each of the five 200 ns MD simulations were cut into 1 ns non-overlapping, consecutive segments. For each PDZ domains, every possible pairs of 1 ns segments were compared by determining the absolute difference of the overall binding site fluctuation (Q) measure calculated for the two different segments. These absolute difference values have been collected in a distribution that was used as a reference background distribution to describe how well Q was converged.

Since even for the same PDZ domain, the values of Q calculated for different MD conformational ensembles are never exactly the same, it should be assessed whether the differences we see between different PDZ domains are significant or within thermal noise. Therefore the spread of the reference background distribution was analysed to calculate p-values of the absolute Q differences found between different PDZ domains.

Our results showed that the differences detected between the overall binding site fluctuations of different PDZ domains are statistically highly significant. For example, the Q difference between InaD PDZ1 and GRIP1 PDZ7 has a p-value of 0.0212, while the significance of the difference found between InaD PDZ1 and PTP-BL PDZ2 is a p-value of 0.002. Additional examples are the differences between Dvl2 PDZ and PTP-BL PDZ2 or Erbin PDZ that have p-values of 0.0045 and 0.0015, respectively, or the difference between GRIP1 PDZ7 and Erbin PDZ that has a p-value of 0.022.

As the above is estimated from 1 ns simulation segments, this could be considered a lower limit as longer simulation times (for example from the whole 200 ns) period are likely to exhibit greater convergence.

Assessment of convergence.

To check whether the conformational sampling was sufficient in the 200 ns simulations, for each PDZ domains, the root mean square inner product (RMSIP) (also referred to as subspace overlap) between the two halves of the simulation was calculated. Previous studies applying such analysis have reported that RMSIP values between 0.5-0.7 can be considered representative of adequate convergence [1].

For PTP-BL PDZ2, the RMSIP measure calculated for the whole domain (all Cα-atoms) between the first 10 principal component vectors, was 0.53. Furthermore, the RMSIP between the binding pocket residues only, was 0.65, indicating adequate convergence. Since the fluctuation analysis were performed on the binding sites, we were mostly interested in the convergence of conformational sampling of the the binding site residues. The first 10 principal components for which the RMSIP overlap were calculated describe the 91.4 % of the variance of conformational sampling of the PTP-BL PDZ2 binding pocket.

The RMSIP measure calculated between the two halves of the 200 ns simulation of the other four PDZ peptide-binding sites also show sufficient convergence. The simulations of the GRIP1 PDZ7 domain, InaD PDZ1 domain, Dvl2 PDZ domain and Erbin PDZ domain provided an RMSIP of 0.69, 0.67, 0.6 and 0.59, respectively.

One can also use the RMSIP measure to study the conformational overlap between different simulations. The RMSIP overlaps between the 200 ns simulations of different PDZ domains have been calculated focusing the analysis on the binding site residues only. The results (summarized in Table S1) show that there were considerable overlaps between the subspaces sampled in the different simulations. The reason why these RMSIP values are higher than those found between the two halves of the simulations is probably that in the current analysis 200 ns trajectories were compared (as opposed to 100 ns previously).

The RMSIP values show that the essential subspaces (defined by the first 10 PC vectors) are similar for the five PDZ binding sites. This is not in contradiction to the observations that the five binding sites have different fluctuation properties. As we have shown in our previous paper [2], the fluctuation matrices used to compare protein motions give different, complementary information than the correlation/covariance matrices which form the basis of Principal Component Analysis. There appears to be notable similarity between the correlated motions of binding site residues in the five PDZ domains, however, the extent of fluctuations are considerably different.

Table S1. Sub-space overlap of the first 10 principal components.

	Dvl2 PDZ	GRIP1 PDZ7	PTP-BL PDZ2	Erbin PDZ
InaD PDZ1	0.67	0.74	0.61	0.70
Dvl2 PDZ		0.6	0.71	0.68
GRIP1 PDZ7			0.65	0.62
PTP-BL PDZ2				0.7

It is important to note that even if the RMSIP overlap between two halves of the simulation was 1 (i.e. the theoretical maximum), this would only mean that the two conformational subspaces sampled in the two halves are identical. It would not give, however, any indication about the completeness of the sampling. For example, perfect overlap (RMSIP = 1) could be observed when the simulation consistently samples only a subspace of the biologically relevant conformational space. In fact, no method that uses the simulation trajectory alone, can determine whether the sampling is complete. As a consequence, however long MD simulation one analyses, it is impossible to tell whether the conformational space would get any closer to an experimentally determined conformation in a longer simulation.

The ratoionale behind using 200 ns simulations was that in our previous study [2] we have shown by an alternative convergence analysis approach that 20 ns simulations of PDZ domains provide robust fluctuation matrix patterns suitable for comparative analysis. Thus using here ten times longer, 200 ns, simulations we expected to get adequately converged fluctuation and flexibility patterns.

Kinetic analysis of conformational states

In order to assess whether the conformational clusters defined by the dRMSD structural similarity measure also correspond to metasable states defined by kinetics, we analysed the temporal distributions of the conformations. We compared the average intra-cluster relaxation time and average inter-cluster transition time for the clusters defined by the k-mean clustering described in the main manuscript. The former was calculated as the average of intra-cluster relaxation times of all simulation frames, where the "intra-cluster relaxation time" of a frame was defined as the time difference from the closest subsequent frame belonging to the same structural cluster. Similarly, the latter was calculated as the average of inter-cluster transition times of all simulation frames, where the "inter-cluster transition time" of a frame was defined as the time difference from the closest subsequent frame belonging to a different structural cluster.

For the two PDZ domains for which distinct conformational clusters were found with k-mean clustering (Dvl2 PDZ and InaD PDZ1), the average inter-cluster transition time was much longer than the average intra-cluster relaxation time. In case of Dvl2 PDZ, the average inter-cluster transition time was 7030 ps, while the average intra-cluster relaxation time was 100 ps. In case of InaD PDZ1, the average inter-cluster transition time was 14100 ps, while the average intra-cluster relaxation time was 100 ps.

Holo PTP-BL PDZ2 Simulation

As mentioned in the main text, we assessed the exploration of the conformational space by the PTP-BL PDZ2 in complex with the APC peptide (starting from PDB code 1VJ6). We analyzed the mean dRMSD dissimilarity between the experimentally determined ligand-bound conformation and the most similar, 10 most similar, 100 most similar and 200 most similar snapshots of the holo simulation. As discussed in the manuscript, these results can be summarized with the help of Q(k)-values (see exact definition in Methods). We found that the Q-values calculated from the holo simulation are slightly lower than those obtained from the apo simulation of PTP-BL PDZ2 (see the Q-values in main text, Table T4). This further indicates that there are regions in the conformational space explored by the holo complex that are not visited in the apo simulation. It therefore lends further support to the idea that the induced fit mechanism plays a role in the binding of the APC peptide to PTP-BL PDZ2 domain.

Table S2. Q-values for the holo complex of PTP-BL PDZ2 with APC peptide.

Holo Complex	$Q^{(1)}$ (Å)	$Q^{(10)}$ (Å)	$Q^{(100)}$ (Å)	$Q^{(200)}$ (Å)
PTP-BL PDZ2 + APC	0.35	0.37	0.40	0.42

References

1. Laberge M, Yonetani T (2008) Molecular dynamics simulations of hemoglobin A in different states and bound to DPG: Effector-linked perturbation of tertiary conformations and HbA concerted dynamics. Biophys J 94: 2737-2751.
2. Münz M, Lyngsø R, Hein J, Biggin PC (2010) Dynamics-based alignment of proteins: An alternative approach to quantify dynamic similarity. BMC Bioinformatics 11: 118.
